# Sentinel lymph node detection in oral cancer: a within-patient comparison between [^99m^Tc]Tc-tilmanocept and [^99m^Tc]Tc-nanocolloid

**DOI:** 10.1007/s00259-020-04984-8

**Published:** 2020-08-25

**Authors:** Inne J. den Toom, Rutger Mahieu, Rob van Rooij, Robert J. J. van Es, Monique G. G. Hobbelink, Gerard C. Krijger, Bernard M. Tijink, Bart de Keizer, Remco de Bree

**Affiliations:** 1 Department of Head and Neck Surgical Oncology, University Medical Center Utrecht, the Netherlands, Heidelberglaan 100, 3584 CX Utrecht, Netherlands; 2 Department of Radiology and Nuclear Medicine, University Medical Center Utrecht, the Netherlands, Heidelberglaan 100, 3584 CX Utrecht, Netherlands

**Keywords:** Oral cancer, Sentinel lymph nodes, Lymphatic metastasis, Lymphoscintigraphy, [^99m^Tc]Tc-tilmanocept

## Abstract

**Purpose:**

Sentinel lymph node (SLN) biopsy has proven to reliably stage the clinically negative neck in early-stage oral squamous cell carcinoma (OSCC). [^99m^Tc]Tc-tilmanocept may be of benefit in OSCC with complex lymphatic drainage patterns and close spatial relation to SLNs.

**Methods:**

A prospective within-patient evaluation study was designed to compare [^99m^Tc]Tc-tilmanocept with [^99m^Tc]Tc-nanocolloid for SLN detection. A total of 20 patients with early-stage OSCC were included, who underwent lymphoscintigraphy with both tracers. Both lymphoscintigraphic images of each patient were evaluated for SLN detection and radiotracer distribution at 2–4 h post-injection.

**Results:**

The injection site’s remaining radioactivity was significantly lower for [^99m^Tc]Tc-tilmanocept (29.9%), compared with [^99m^Tc]Tc-nanocolloid (60.9%; *p* < 0.001). Radioactive uptake in SLNs was significantly lower for [^99m^Tc]Tc-tilmanocept (1.95%) compared with [^99m^Tc]Tc-nanocolloid (3.16%; *p* = 0.010). No significant difference was seen in SLN to injection site ratio in radioactivity between [^99m^Tc]Tc-tilmanocept (0.066) and [^99m^Tc]Tc-nanocolloid (0.054; *p* = 0.232). A median of 3.0 and 2.5 SLNs were identified with [^99m^Tc]Tc-tilmanocept and [^99m^Tc]Tc-nanocolloid, respectively (*p* = 0.297). Radioactive uptake in higher echelon nodes was not significantly different between [^99m^Tc]Tc-tilmanocept (0.57%) and [^99m^Tc]Tc-nanocolloid (0.86%) (*p* = 0.052). A median of 2.0 and 2.5 higher echelon nodes was identified with [^99m^Tc]Tc-tilmanocept and [^99m^Tc]Tc-nanocolloid, respectively (*p* = 0.083).

**Conclusion:**

[^99m^Tc]Tc-tilmanocept had a higher injection site clearance, but at the same time a lower uptake in the SLN, resulting in an SLN to injection site ratio, which was not significantly different from [^99m^Tc]Tc-nanocolloid. The relatively low-radioactive uptake in SLNs of [^99m^Tc]Tc-tilmanocept may limit intraoperative detection of SLNs, but can be overcome by a higher injection dose.

**Electronic supplementary material:**

The online version of this article (10.1007/s00259-020-04984-8) contains supplementary material, which is available to authorized users.

## Introduction

The sentinel lymph node biopsy (SLNB) procedure is a diagnostic staging method that is applied in a variety of tumour types, including oral squamous cell carcinoma (OSCC). The procedure aims to identify the first draining lymph nodes, the ‘sentinel lymph nodes’ (SLN), which are most likely to harbour metastases. The histopathological status of the SLN should reflect the histopathological status of the rest of the nodal basin, and additional treatment of the nodal basin (e.g. surgery or radiotherapy) should only be performed in case of metastatic involvement of the SLN. So far, the routine procedure consists of preoperative peritumoural injection of a 99-m technetium ([^99m^Tc])-labelled colloid followed by dynamic and static lymphoscintigraphy using planar and single-photon emission computed tomography (SPECT) imaging [1–3]. Intraoperative detection is possible using a portable gamma probe.

**Fig. 1:**
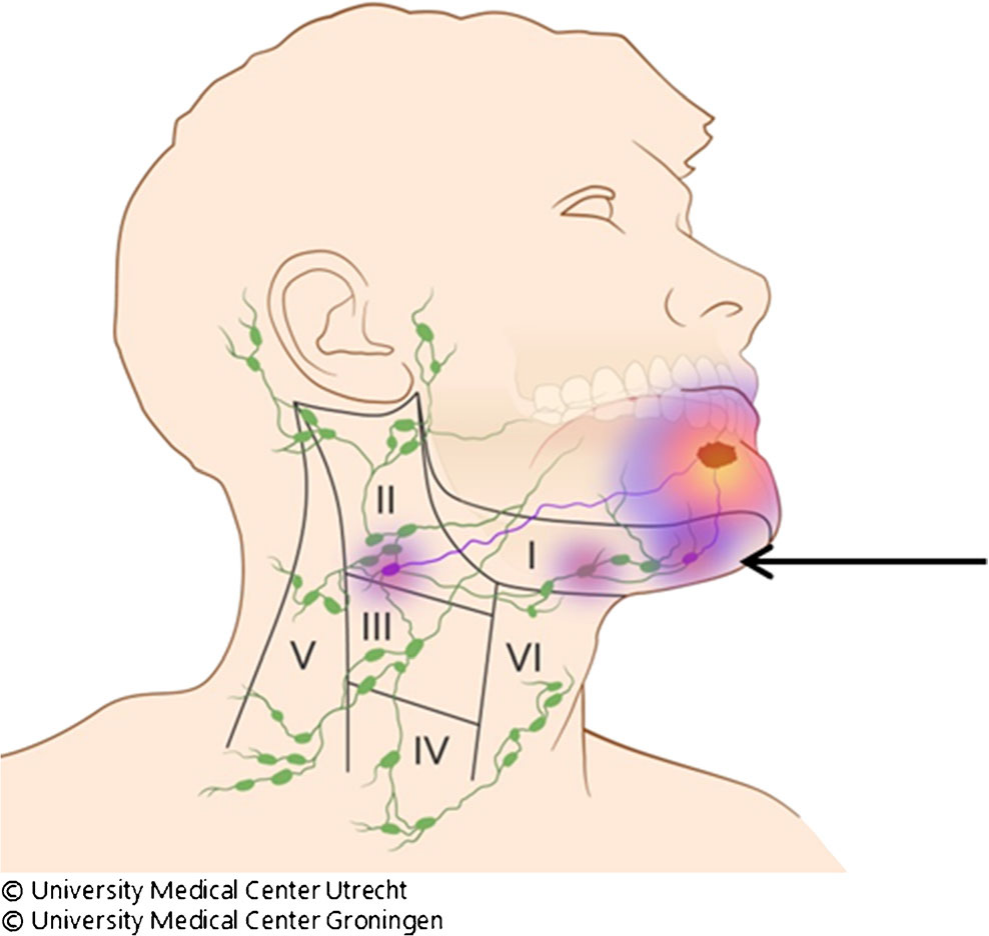
Shine through phenomenon. Radiation flare of the primary tumour overshines the hotspot of sentinel lymph node in close proximity to the primary tumour (arrow)

It has been demonstrated that by using this approach, the SLNB procedure reliably stages the clinically negative neck (cN0) in early-stage OSCC with a sensitivity of 87% and a negative predictive value of 94% in the most recent meta-analysis [4]. However, one of the most frequently mentioned difficulties of this procedure occurs when the injection site around the primary tumour produces a large hotspot on lymphoscintigraphy, possibly hiding SLN(s) in close proximity of the primary tumour, usually referred as ‘shine through’ phenomenon (Fig. 1). This phenomenon is particularly evident in floor of mouth tumours, and multiple studies demonstrated a (significantly) lower accuracy of the SLNB procedure in floor of mouth tumours compared with other tumour locations in the oral cavity [5–8]. Some authors even advocate adding a superselective level I resection in these cases [9]. Secondly, on lymphoscintigraphy, it is often difficult to differentiate hotspots between SLNs and second echelon nodes [10]. As a result, second echelon lymph nodes may erroneously be considered SLNs, resulting in an unnecessary extension of the surgical procedure.

**Fig. 2:**
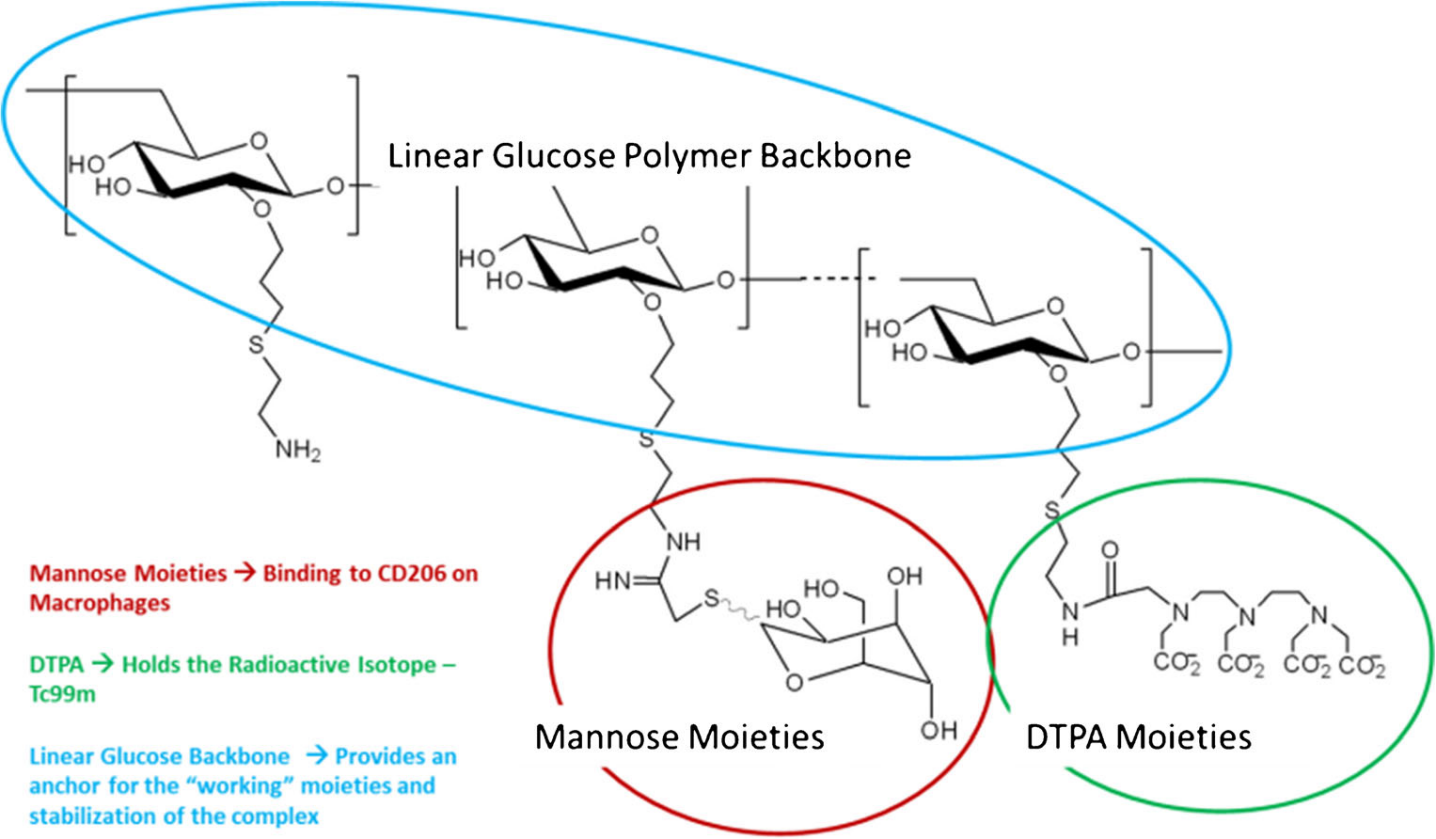
[^99m^Tc]Tc-tilmanocept (Lymphoseek) structure and functional elements

A new radioactive agent, [^99m^Tc]Tc-tilmanocept (Lymphoseek®, Navidea Biopharmaceuticals, Inc.), has been specifically designed for SLN identification and is registered for this purpose in both the USA and Europe. [^99m^Tc]Tc-tilmanocept is a small-sized receptor-targeted (CD206) sentinel lymph node detection agent (Fig. 2) [11]. Due to its proposed rapid clearance from the injection site, rapid uptake and high retention within the SLN, and low uptake by the remaining (higher echelon) lymph nodes, [^99m^Tc]Tc-tilmanocept may particularly be of benefit in floor of mouth tumours and other head and neck tumours with complex drainage patterns and close spatial relation to the SLN [12, 13]. A multicentre validation study using [^99m^Tc]Tc-tilmanocept for SLNB in head and neck squamous cell carcinoma showed an SLN identification rate of 97.6%, a false-negative rate of 2.56%, and a negative predictive value of 97.8% [14]. Of note, these high figures were also obtained in floor of mouth cancers, which strengthened the idea that [^99m^Tc]Tc-tilmanocept may diminish the shine through effect and improve the SLN detection rate for this subsite.

In Europe, [^99m^Tc]Tc-nanocolloid is the most frequently used radiocolloid for SLN mapping. So far, there are no studies performed comparing head to head [^99m^Tc]Tc-tilmanocept with [^99m^Tc]Tc-nanocolloid.

The aim of the present study is to investigate the injection site clearance and uptake in SLN(s) of [^99m^Tc]Tc-tilmanocept in comparison with a standard [^99m^Tc]Tc-nanocolloid by means of lymphoscintigraphy in early-stage oral cancer patients.

## Material and methods

A monocentre prospective within-patient evaluation study was designed in order to compare [^99m^Tc]Tc-tilmanocept with our routinely used [^99m^Tc]Tc-nanocolloid tracer, in terms of SLN visualization, injection site clearance, and uptake in SLN(s). This study was approved by the medical ethical review board of the University Medical Center Utrecht (NL58099.041.17).

All patients had an early-stage cT1-2N0M0 OSCC (TNM Staging AJCC UICC 8th Edition). Clinical nodal staging was confirmed by at least ultrasound and, in case of suspicious lymph nodes, ultrasound-guided fine-needle aspiration cytology. In most cases, MRI was conducted as well, as part of clinical staging.

Patients with a history of neck dissection, neck irradiation, or gross injury to the neck that would hamper surgical dissection of SLNs were excluded from this study. Besides, patients with a history of head and neck malignancies in the last 5 years were excluded as well.

**Fig. 3:**
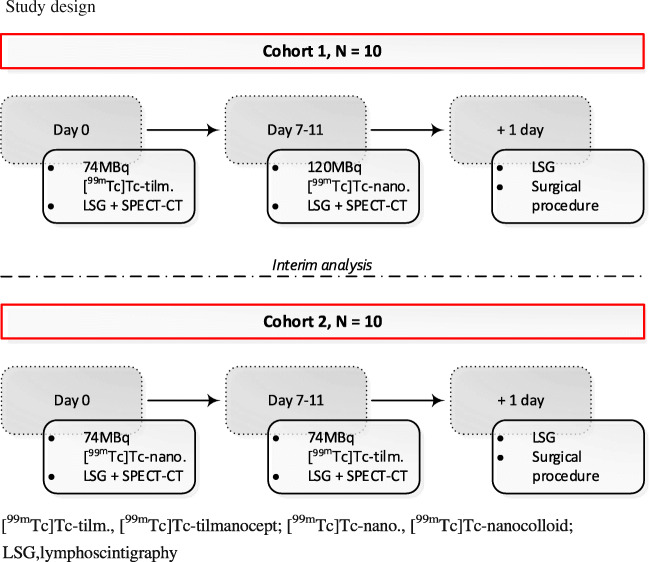
Study design. [^99m^Tc]Tc-tilm., [^99m^Tc]Tc-tilmanocept; [^99m^Tc]Tc-nano., [^99m^Tc]Tc-nanocolloid; LSG, lymphoscintigraphy

This study consisted of 2 groups containing 10 patients each (Fig. 3). In the first group (cohort 1), 50 μg of [^99m^Tc]Tc-labelled tilmanocept (74 MBq in 0.4 mL) was prepared according to manufacturer’s instructions. All tracers were administered in 4 peritumoural injections of 0.1 mL, followed by lymphoscintigraphy. Four to 11 days later, these 10 patients subsequently underwent a [^99m^Tc]Tc-nanocolloid (routine dose 120 MBq) lymphoscintigraphy. After the first cohort, interim analysis was carried out before continuing with the second cohort.

In cohort 2, tracers were administered in opposite order; first, 74 MBq [^99m^Tc]Tc-nanocolloid, followed by 74 MBq [^99m^Tc]Tc-tilmanocept. In both cohorts, the same imaging protocol was applied.

In an effort to administer both tracers at the same injection spots, photographic images were made of the peritumoural injections with consent of patients. Following injection of the second radio-agent, the same imaging protocol was applied. Patients reported their pain scores during the injection procedure for both tracers using the Numeric Pain Rating Scale (NPRS) [15].

### Imaging protocol

Directly, post-injection planar images were acquired in dynamic mode (128 × 128 matrix, 20 frames of 1 min) in anterior-posterior projection followed by static mode (256 × 256 matrix, during 4 min) in anterior-posterior and lateral projections (30 min and 2 h post-injection), on a Siemens Symbia T16 SPECT-CT scanner, using ‘low- and medium energy’ (LME) collimators to limit septal penetration (reducing shine through) [16]. In addition to the planar imaging 2 h post-injection, SPECT-CT scans were acquired on a 128 × 128 matrix (pixel spacing, 3.9 × 3.9 mm), with 128 angles, 20 s per projection, over a non-circular 360° orbit (CT: 110 kV, 40 mAs eff., 16 × 1.2 mm). SPECT images were reconstructed using clinical reconstruction software (Siemens Flash3D), with attenuation and scatter correction (6 iterations, 8 subsets, 5-mm Gaussian filter). Additionally, quantitative SPECT reconstructions were generated using the Utrecht Monte Carlo System (UMCS), a dedicated SPECT reconstructor [17, 18] which includes Monte Carlo modelling of scatter and collimator-detector interactions. During lymphoscintigraphy, a source with known radioactivity was scanned in the same frame as the patient, acting as a verification of quantitative accuracy.

### Intraoperative detection and histology

Intraoperative detection of SLN(s) was performed using a portable gamma probe, according to standard protocol [3]. The last injected radio-agent was leading to identify SLNs during surgery. In the present study, no superselective neck dissection of the preglandular triangle of level I was performed in floor of mouth tumours. All harvested nodes were histologically examined for metastasis using step serial sectioning (intervals of 150 μm) with haematoxylin-eosin and pan-cytokeratin antibody (AE 1/3) staining at each level.

### Evaluation of images

Paired images of both tracers were evaluated regarding similarity of depicted draining lymph node basins, the number and location of SLNs, and their histopathology. Furthermore, the amount of radioactivity that resided in the injection site, SLNs, higher echelon nodes, and reference source were measured from quantitative SPECT-CT images, acquired 2 h post-injection.

**Fig. 4:**
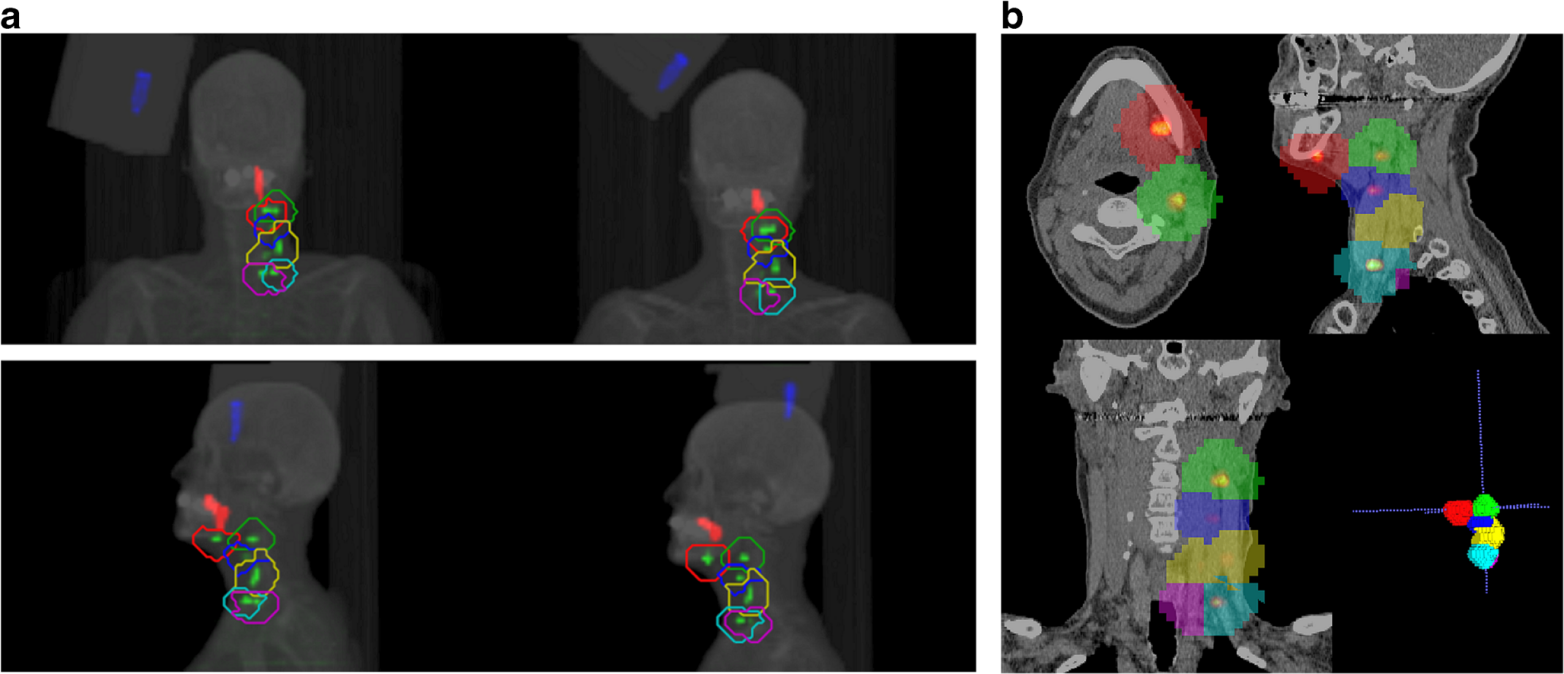
**a** Algorithmic-defined VOIs for all hotspots within the scanned area for both [^99m^Tc]Tc-tilmanocept and [^99m^Tc]Tc-nanocolloid. Summed intensity projections of SPECT reconstructions of the same patient, injected with either [^99m^Tc]Tc-tilmanocept (left) or [^99m^Tc]Tc-nanocolloid (right). Injection site: *Red hotspot*. Reference source: *Blue hotspot.* ‘Hot’ lymph nodes: *Green hotspots with coloured VOIs*. **b** Verification of VOIs containing ‘hot’ lymph nodes using 3D segmentation software (ITK-SNAP). Sentinel lymph nodes: Red and green VOI. Higher echelon nodes: blue, yellow, turquoise, and purple VOI

Volumes of interest (VOIs) around the injection site, SLNs and the reference source were automatically defined using in-house developed software, adopting a local peak finding algorithm and watershed segmentation [19] (Fig. 4a). The VOIs were manually validated with 3D segmentation software ITK-SNAP [20] (Fig. 4b).

All quantitative results of VOI measurements are presented as percentages of the amount of injected radioactivity. The remaining radioactivity outside of the VOIs but within field of view of the SPECT acquisition was regarded to be 99-m technetium located outside the (S)LNs, injection site, or reference source and was further addressed as background radioactivity. Since the measured cumulative background radioactivity is strongly dependent on the volume of the patient within the field of view of SPECT acquisition, the background activity is also presented in terms of standardized uptake value (SUV), analogous to PET (i.e. average measured activity concentration in background, divided by the average activity concentration in the entire patient, based on body mass).

For qualitative evaluation of [^99m^Tc]Tc-nanocolloid and [^99m^Tc]Tc-tilmanocept lymphoscintigraphy, images of each subject for both tracers were blinded and scored by 2 head and neck surgeons and 2 nuclear medicine physicians. Per image, every hotspot was classified as SLN using a 3-point scale (yes, potential, no). Afterwards, every ‘potential’ scored SLN was eventually dichotomized into ‘yes’ or ‘no’ by the observers, based on their advice to surgically harvest the concerning lymph node. Besides, all observers rated the difficulty for reviewing the images (i.e. easy, moderate, hard). Interobserver variability regarding the selected SLNs between observers was assessed.

Ultimately, data from qualitative analyses were matched with quantitative results of corresponding VOIs and correlated with intraoperative and pathological findings of the harvested (S)LNs.

### Statistical analyses

All data was analyzed with professional statistics software (IBM SPSS Statistics version 25.0). Data is expressed as mean ± SD for parametric continuous variables and as median for nonparametric continuous variables. Number of cases and percentages are presented as categorical variables. All quantitative results of VOI measurements are presented as percentages of the amount of injected radioactivity.

To compare the amount of radioactivity in the injection site, SLNs, higher echelon nodes and background between [^99m^Tc]Tc-tilmanocept and [^99m^Tc]Tc-nanocolloid, and paired samples *t* tests were applied for parametric variables, while Wilcoxon signed-rank tests were applied for nonparametric variables. To compare the ‘SLN to injection site ratio’ in radioactivity between [^99m^Tc]Tc-nanocolloid and [^99m^Tc]Tc-tilmanocept, a Wilcoxon signed-rank test was applied.

To determine interobserver variability regarding selected SLNs between observers for both [^99m^Tc]Tc-nanocolloid and [^99m^Tc]Tc-tilmanocept lymphoscintigraphic images, Fleiss’ kappa statistics were applied [21]. Finally, to compare the rated difficulty for reviewing [^99m^Tc]Tc-nanocolloid and [^99m^Tc]Tc-tilmanocept lymphoscintigraphic images, McNemar tests were applied.

A *p* value < 0.05 was regarded as statistically significant.

## Results

Characteristics of the 20 patients and tumours are listed in Table 1. The oral tongue was the most affected tumour location. In 5 (25%) cases, the floor of mouth was involved. In total, 49 SLNs were harvested (median 2), of which 12 (24%) showed metastasis. These 12 positive SLNs were harvested from 7 patients, making 35% (7/20) of our study population positive for lymphatic metastasis. Distribution of hotspots and SLNs per tracer per patient is given in the supplementary data 1.

**Table 1:** Patient characteristics

Characteristics	Overall (%)
Patients, *n* (%)	20 (100)
Gender, *n* (%)	
Male	13 (65)
Female	07 (35)
Median age (year) (range)	63 (39–77)
Tumour location, *n* (%)	
Tongue	14 (70)
Floor of mouth	05 (25)
Lower gum	01 (5)
Clinical T stage, *n* (%)*	
T1	09 (45)
T2	11 (55)
Pathology primary tumour	
Diameter (mm) (range)	19 (6–44)
Depth of invasion (mm) (range)	06 (1–13)
Pathology sentinel lymph nodes	
Negative	37 (76)
Positive	12 (24)
Median-harvested SLNs (range)	02 (1–5)
Number of SLN-positive patients	07 (35)

*T stage according to 8th AJCC TNM classification

### Quantitative analyses (Table 2)

The radioactivity remaining in the injection site was significantly lower for [^99m^Tc]Tc-tilmanocept (29.9%; SD ± 7.6), compared with [^99m^Tc]Tc-nanocolloid (60.9%; SD ± 16.1) (*p* < 0.001).

**Table 2:** Quantitative analyses

	[^99m^Tc]Tc-tilmanocept	[^99m^Tc]Tc-nanocolloid	*p* value
Radioactivity remaining in injection site	29.9%; SD ± 7.6 (range 17.10–43.95)	60.9%; SD ± 16.1 (range 30.26–89.58)	< 0.001
Uptake in SLNs	1.95%; IQR ± 2.6 (range 0.21–6.80)	3.16%; IQR ± 3.9 (range 0.04–11.90)	0.010
SLN to injection site ratio	0.066; IQR ± 0.1 (range 0.001–0.20)	0.054; IQR ± 0.07 (range 0.001–0.22)	0.232
Number of SLNs	3.0; IQR ± 2 (range 0–4)	2.5; IQR ± 1 (range 1–5)	0.297
Number of higher echelon nodes	2.0; IQR ± 2 (range 0–5)	2.5; IQR ± 3 (range 0–6)	0.083
Uptake in higher echelon nodes	0.57%: IQR ± 1.64 (range 0.001–7.15)	0.86%: IQR ± 2.17 (range 0.001–6.95)	0.052
Background activity	2.23%; IQR ± 2.01 (range 0.93–5.76)	0.41%; IQR ± 0.96 (range 0.01–1.55)	< 0.001
Pain score (NPRS)	3.0; IQR ± 3 (range 0–8)	2.0; IQR ± 4 (range 0–8)	0.041

SD, standard deviation; IQR, interquartile range; SLN, sentinel lymph node

The radioactive uptake in SLNs was significantly lower for [^99m^Tc]Tc-tilmanocept compared with [^99m^Tc]Tc-nanocolloid (1.95% vs. 3.16% respectively, *p* = 0.010). The SLN to injection site ratio between [^99m^Tc]Tc-tilmanocept (0.066) and [^99m^Tc]Tc-nanocolloid (0.054) was not statistically different (*p* = 0.232).

In 20 patients, a median of 3.0 and 2.5 SLNs were identified with [^99m^Tc]Tc-tilmanocept and [^99m^ Tc]Tc-nanocolloid, respectively (*p* = 0.297).

The number of higher echelon nodes did not differ significantly between both tracers with a median of 2.0 in the [^99m^Tc]Tc-tilmanocept cohort and 2.5 in the [^99m^Tc]Tc-nanocolloid group (*p* = 0.083). [^99m^Tc]Tc-tilmanocept showed less radioactive uptake in higher echelon nodes in comparison with the [^99m^Tc]Tc-nanocolloid group, although not statistically significant (0.57% vs. 0.86% respectively, *p* = 0.052).

[^99m^Tc]Tc-tilmanocept showed a higher background radioactivity in comparison with [^99m^Tc]Tc-nanocolloid (2.23% vs. 0.41% in field of view of the SPECT, *p* < 0.001. SUV: 0.132 vs. 0.018, *p* < 0.001).

A median pain score (NPRS) of 3.0 (range 0–8) was reported for [^99m^Tc]Tc-tilmanocept compared with 2.0 (range 0–8) for [^99m^Tc]Tc-nanocolloid (*p* = 0.041).

### Qualitative analyses

Interobserver agreement regarding selection of SLNs with a 3-point scale using the Fleiss kappa statistics showed substantial agreement for both [^99m^Tc]Tc-tilmanocept and [^99m^Tc]Tc-nanocolloid (*κ* = 0.677 [95% CI 0.619–0.735] vs. *κ* = 0.725 [95% CI 0.668–0.782] respectively, not significantly different). When dichotomizing, both tracers reached excellent agreement with an equal Fleiss’ kappa (*κ* = 0.885 [95% CI 0.804–0.966] for [^99m^Tc]Tc-tilmanocept and *κ* = 0.885 [95% CI 0.806–0.963] for [^99m^Tc]Tc-nanocolloid).

[^99m^Tc]Tc-tilmanocept scans were categorized scored as easy (6×), moderate (10×), and hard (4×), whereas [^99m^Tc]Tc-nanocolloid was ranked as easy (6×), moderate (9×), and hard (5×) (McNemar test, *p* = 0.80).

No serious adverse events or allergic reactions were reported in our study population.

## Discussion

The present study is the first within-patient evaluation comparing [^99m^Tc]Tc-tilmanocept with [^99m^Tc]Tc-nanocolloid. We showed a significantly higher injection site clearance for [^99m^Tc]Tc-tilmanocept but also a significantly lower uptake in the SLN in comparison with [^99m^Tc]Tc-nanocolloid. No significant difference was seen in SLN to injection site ratio. There was an excellent interobserver agreement for both [^99m^Tc]Tc-tilmanocept and [^99m^Tc]Tc-nanocolloid. Thereby, difficulty of scan interpretation was equal for both tracers.

Currently, there are no other within-patient evaluation studies comparing [^99m^Tc]Tc-tilmanocept to another radioactive tracer. Only one RCT so far has been published by Unkart et al., who presented a trial of 57 breast cancer patients comparing [^99m^Tc]Tc-tilmanocept with [^99m^Tc]Tc-sulphur colloid regarding pain after injection of both tracers [22]. They showed a higher pain sensation in the first 3 min after injection of [^99m^Tc]Tc-sulphur colloid compared with [^99m^Tc]Tc-tilmanocept. In contrast, in our study, a higher pain score was found for [^99m^Tc]Tc-tilmanocept as compared with [^99m^Tc]Tc-nanocolloid, regardless whether [^99m^Tc]Tc-tilmanocept was injected as first or second tracer. However, our study size is small and the clinical relevance of a difference of 1 point (median 2.0 vs. 3.0) is questionable.

Additionally, Unkart et al. found no statistical differences in breast cancer patients concerning number of hotspots, number of removed SLNs, time to surgical removal, or number of blue nodes for [^99m^Tc]Tc-Tilmanocept compared with [^99m^Tc]Tc-sulphur colloid [23]. However, this study was not especially designed for analyzing differences regarding SLN identification. Randomizing patients for either the one or the other tracer did not clearly clarify discrepancies between both tracers with respect to drainage patterns due to a high variability in lymphatic drainage per patient, especially in complex lymphatic regions. Therefore, it is our opinion that a within-patient study design is superior to reveal characteristics regarding lymphatic drainage patterns of both tracers.

As already mentioned in the ‘Introduction’, [^99m^Tc]Tc-tilmanocept was specifically designed for SLN identification, providing characteristics that could be of potential value in complex lymphatic regions, as is the case in OSCC. Our data clearly underlines its theoretical effect of a more rapid clearance of the radioactivity from the injection site due to its smaller molecular size. This may benefit SLN detection, particularly in situations with close spatial relation between injection site and SLNs, which is especially the case in floor of mouth tumours. Using [^99m^Tc]Tc-tilmanocept, Agrawal et al. supported this theory with an impressively low false-negative rate of 2.56% for SLNB in OSCC, which was also found in FOM tumours [14]. In that study, however, a complementary neck dissection in the same session was performed as validation method (reference standard) for the SLNB procedure. However, micrometastases remain undetected in up to 15% of routinely processed neck dissection specimens [24, 25]. Therefore, in case of a negative SLNB, a wait-and-scan approach should be considered the best gold standard [26]. As a consequence, further studies with long-term follow-up are needed to investigate the efficacy of [^99m^Tc]Tc-tilmanocept for detection of occult metastases.

In our study, a higher percentage of radioactivity in background was seen for [^99m^Tc]Tc-tilmanocept compared with [^99m^Tc]Tc-nanocolloid. One possible explanation could be the smaller molecular diameter of 7 nm, which enhances diffusion into lymphatic channels as well as blood capillaries. As stated by Ellner et al., [^99m^Tc]Tc-tilmanocept showed a percentage of injected dose below 2.6% for the liver, kidney, bladder, and head [27]. Although the background radioactivity for [^99m^Tc]Tc-tilmanocept was still marginal (2.23%; SUV 0.132), it explains the residual distribution of [^99m^Tc]Tc-tilmanocept in the presence of a lower radioactivity residing in both the injection site, as well as in the lymph nodes.

One of our study limitations is the difference in amount of radioactivity between both tracers in the first 10 patients: 74 MBq [^99m^Tc]Tc-tilmanocept vs. 120 MBq [^99m^Tc]Tc-nanocolloid, respectively. [^99m^Tc]Tc-tilmanocept was approved by the FDA (Food and Drug Administration) and EMA (European Medicines Agency) for identification of SLNs using 74 MBq in a 2-day protocol. In our institution, SLNB is routinely performed with 120 MBq [^99m^Tc]Tc-nanocolloid. Because the first 10 patients were surgically treated based on [^99m^Tc]Tc-nanocolloid, they received this routinely used amount of radioactivity to safely perform SLNB. This difference was corrected during quantitative analysis by correlating measured radioactivity in the VOIs to the radioactive-dose injected. In the second 10 patients, [^99m^Tc]Tc-tilmanocept was leading for SLNB procedure, and therefore, the amount of radioactivity could be equalized for both tracers (74 MBq). Another limitation is the impossibility of comparing hotspots at different time points post-injection. Due to the impossibility of performing attenuation correction on planar lymphoscintigraphy, we unfortunately could not reliably compare SLN visualization at different time points due to different imaging modalities. Intensity of hotspots could easily be under- or overestimated based on physiological structures in near surroundings (e.g. mandible). On planar lymphoscintigraphy, only anterior-posterior or oblique images could be used. This impedes us from differentiating and analyzing hotspots located in the same plane. Therefore, we opted to perform only quantitative analysis based on SPECT-CT.

In some patients for whom [^99m^Tc]Tc-tilmanocept was leading to identify SLNs during surgery, it proved challenging to accurately locate SLNs due to a scarce of activity on the second day, which was considered a drawback by the surgeon. This may be due to the relatively low-radioactive uptake in SLNs of [^99m^Tc]Tc-tilmanocept that was seen in our population. As the injected activity was lower than what was used in [^99m^Tc]Tc-nanocolloid SLNB (74 vs. 120 MBq) with also lower uptake in SLNs (3.16% vs. 1.95%) this resulted in less activity in SLNs in SLNB with [^99m^Tc]Tc-tilmanocept, on average 1.4 MBq vs. 3.8 MBq at time of SLN scintigraphy. Vidal-Sicart et al. faced similar challenges during intraoperative localisation of SLNs using [^99m^Tc]Tc-tilmanocept, which can probably be overcome by a higher injection dose of [^99m^Tc]Tc-tilmanocept [13].

In conclusion, our results suggest that [^99m^Tc]Tc-tilmanocept had a higher injection site clearance, but at the same time a lower uptake in the SLN, resulting in an SLN to injection site ratio, which was not significantly different from [^99m^Tc]Tc-nanocolloid. The relatively low-radioactive uptake in SLNs of [^99m^Tc]Tc-tilmanocept may limit intraoperative detection of SLNs, but might be overcome by a higher injection dose.

## Electronic supplementary material

ESM 1 (DOCX 21 kb)
