# Supplementary material for: Sentinel lymph node detection in oral cancer: a within-patient comparison between [99mTc]Tc-tilmanocept and [99mTc]Tc-nanocolloid
Source: Eur J Nucl Med Mol Imaging. 2020 Aug 25;48(3):851–8. doi: 10.1007/s00259-020-04984-8 (PMC8036184; doi:10.1007/s00259-020-04984-8)
Supplement: Supplementary file 1 — (DOCX 21 kb) [file 259_2020_4984_MOESM1_ESM.docx]

Supplementary data 1: Hotspots and SLN distribution per tracer and per patient.

Note: all italic levels are found with [^99m^Tc]Tc-tilmanocept as radioactive agent.

| **Patient** | **cT stage** | **Tumour location** | **Side** | **Hotspots**  **Agent 1** | | **Hotspots**  **Agent 2** | | **Harvested SLNs peroperatively** | | **Pathology** |
| --- | --- | --- | --- | --- | --- | --- | --- | --- | --- | --- |
| 1 | T2 | Tongue | Left | *IB* | *Left* | IB | Left | IB | Left | Positive |
|  |  |  |  | *II* | *Left* | II | Left | IIA | Left | Negative |
|  |  |  |  | *III* | *Left* | III | Left |  |  |  |
| 2 | T1 | Tongue | Left | *II* | *Left* | II | Left | IIA | Left | Negative |
|  |  |  |  | *II* | *Left* | II | Left | IIA | Left | Negative |
|  |  |  |  |  |  |  |  | IIB | Left | Negative |
|  |  |  |  |  |  |  |  | III | Left | Negative |
| 3 | T2 | Tongue | Right | *II* | *Right* | II | Right | IIA | Right | Negative |
|  |  |  |  |  |  |  |  | IIA | Right | Negative |
| 4 | T1 | Floor of mouth | Right | *IA* | *Right* | IA | Right | IA | Right | Negative |
|  |  |  |  | *IB* | *Right* | IB | Right | IB | Right | Negative |
|  |  |  |  | *IB* | *Left* | III | Right | III | Right | Negative |
|  |  |  |  |  |  | III | Left |  |  |  |
| 5 | T1 | Floor of mouth | Midline | *IB* | *Right* | IB | Right | IB | Right | Negative |
|  |  |  |  | *IB* | *Right* | IB | Left |  |  |  |
|  |  |  |  | *III* | *Right* | III | Right |  |  |  |
|  |  |  |  | *III* | *Right* | III | Left |  |  |  |
| 6 | T2 | Tongue | Right | *II* | *Right* | II | Right | IIB | Right | Negative |
|  |  |  |  |  |  |  |  | IIB | Right | Negative |
| 7 | T2 | Tongue | Right | *II* | *Right* | II | Right | IIB | Right | Positive |
|  |  |  |  | *IB* | *Left* | III | Left | IV | Left | Positive |
|  |  |  |  | *III* | *Left* |  |  | IV | Left | Positive |
| 8 | T1 | Tongue | Left | *IB* | *Left* | II | Left | III | Left | Negative |
|  |  |  |  | *II* | *Left* | II | Left | III | Left | Negative |
| 9 | T2 | Tongue | Right | *I* | *Right* | I | Right | IB | Right | Positive |
|  |  |  |  | *II* | *Right* | II | Right | IIA | Right | Negative |
|  |  |  |  | *III* | *Left* | II | Left | IIA | Left | Negative |
|  |  |  |  | *II* | *Left* | III | Left | III | Left | Negative |
| 10 | T1 | Floor of mouth | Left | *IB* | *Left* | IB | Left | IIA | Left | Negative |
|  |  |  |  |  |  | II | Left | IIA | Left | Negative |
|  |  |  |  |  |  | II | Left |  |  |  |
| 11 | T1 | Floor of mouth | Right | IA | Right | *IA* | *Right* | IIB | Right | Negative |
|  |  |  |  | II | Right | *II* | *Right* | IIB | Right | Negative |
|  |  |  |  | III | Right | *II* | *Left* | IIB | Right | Negative |
|  |  |  |  | II | Left |  |  | IIA | Right | Negative |
|  |  |  |  | IV | Left |  |  | IIB | Left | Negative |
| 12 | T1 | Floor of mouth | Left | IA | Left | *II* | *Left* | IIA | Left | Negative |
|  |  |  |  | III | Left | *III* | *Left* |  |  |  |
|  |  |  |  | III | Right | *III* | *Right* |  |  |  |
| 13 | T2 | Tongue | Right | II | Right | *II* | *Right* | IIA | Right | Negative |
| 14 | T2 | Lower alveolus and gingiva | Left | III | Left | *II* | *Left* | IIA | Left | Positive |
| 15 | T1 | Tongue | Left | IB | Left | *IB* | *Left* | IB | Left | Negative |
|  |  |  |  | IV | Left | *II* | *Left* | IB | Left | Negative |
|  |  |  |  |  |  | *III* | *Left* |  |  |  |
|  |  |  |  |  |  | *IV* | *Left* |  |  |  |
| 16 | T2 | Tongue | Right | II | Right | *II* | *Right* | IIB | Right | Positive |
|  |  |  |  |  |  | *III* | *Right* | III | Right | Positive |
| 17 | T2 | Tongue | Left | II | Left | *II* | *Left* | IIA | Left | Positive |
|  |  |  |  | II | Left | *II* | *Left* | IIA | Left | Positive |
|  |  |  |  |  |  | *II* | *Left* | IIA | Left | Positive |
|  |  |  |  |  |  |  |  | IIB | Left | Negative |
|  |  |  |  |  |  |  |  | IIB | Left | Negative |
| 18 | T2 | Tongue | Left | IA | Left | *IA* | *Left* | IA | Left | Negative |
|  |  |  |  | IB | Left | *IB* | *Left* | IA | Left | Negative |
|  |  |  |  | II | Left | *II* | *Left* | IIB | Left | Negative |
| 19 | T1 | Tongue | Left | IA | Left | *IA* | *Left* | IA | Left | Negative |
|  |  |  |  | II | Left | *II* | *Left* | IIB | Left | Negative |
|  |  |  |  | II | Left |  |  |  |  |  |
| 20 | T2 | Tongue | Left | IB | Right | *II* | *Right* | IB | Left | Positive |
|  |  |  |  | II | Right | *III* | *Right* | III | Left | Negative |
|  |  |  |  | III | Right | *IA* | *Left* |  |  |  |
|  |  |  |  | IB | Left | *IB* | *Left* |  |  |  |
|  |  |  |  | III | Left | *III* | *Left* |  |  |  |
| Note: All *italic levels* are found with [99mTc]Tc-tilmanocept as radioactive agent | | | | | | | | | | |
